# Prevalence of plasma autoantibody against cancer testis antigen NY-ESO-1 in HTLV-1 infected individuals with different clinical status

**DOI:** 10.1186/s12985-017-0802-9

**Published:** 2017-07-17

**Authors:** Yasuo Shiohama, Tadasuke Naito, Toshio Matsuzaki, Reiko Tanaka, Takeaki Tomoyose, Hiroshi Takashima, Takuya Fukushima, Yuetsu Tanaka, Mineki Saito

**Affiliations:** 1 Department of Microbiology, Kawasaki Medical School, 577 Matsushima, Okayama, 701-0192 Japan; 2 Department of Neurology and Geriatrics, Kagoshima University Graduate School of Medical and Dental Sciences, 8-35-1 Sakuragaoka, Kagoshima, 890-8520 Japan; 3 Department of Immunology, Graduate School of Medicine, University of the Ryukyus, 207 Uehara, Okinawa, 903-0215 Japan; 4 Division of Endocrinology, Diabetes and Metabolism, Hematology and Rheumatology, Second Department of Internal Medicine, Graduate School of Medicine, University of the Ryukyus, 207 Uehara, Okinawa, 903-0215 Japan; 5 Laboratory of Hematoimmnology, School of Health Sciences, Faculty of Medicine, University of the Ryukyus, 207 Uehara, Okinawa, 903-0215 Japan; 6 Present Address: Division of Immunogenetics, Department of Immunobiology and Neuroscience, Medical Institute of Bioregulation, Kyushu University, Fukuoka, Japan

**Keywords:** HTLV-1, NY-ESO-1, ATL, HAM/TSP, Monoclonal antibody, ELISA

## Abstract

**Background:**

Detection of specific immune responses against cancer/testis antigen NY-ESO-1 was recently reported in patients with adult T-cell leukemia/lymphoma (ATL) and human T-cell leukemia virus type 1 (HTLV-1)-infected asymptomatic carriers (ACs). However, the relationship of the responses with the HTLV-1 proviral load (PVL) and the levels of viral gene expression remain unclear.

**Findings:**

We measured plasma levels of autoantibodies to NY-ESO-1 immunogenic tumor antigen in HTLV-1-infected individuals with different clinical status, and in healthy controls. Data were compared to *tax* and *HBZ* mRNA levels, and PVL. Plasma anti-NY-ESO-1 antibody was detectable in 13.7% (7/51) of ACs, 29.2% (38/130) of patients with HTLV-1 associated myelopathy/tropical spastic paraparesis (HAM/TSP), and 18.9% (10/53) of patients with ATL. Anti-NY-ESO-1 plasma levels were significantly higher in patients with HAM/TSP than in patients with ATL or ACs. Anti-NY-ESO-1 levels were not associated with PVL or the expression levels of *tax* and *HBZ* mRNA among HTLV-1-infected individuals, regardless of clinical status.

**Conclusions:**

The present results indicate the strong humoral immune response against NY-ESO-1 in natural HTLV-1 infection, irrespective of the clinical status. The higher immunoreactivity against NY-ESO-1 is not simply associated with the levels of both HTLV-1 gene expression and the number of infected cells in vivo. Rather, it might reflect chronic and generalized immune activation in infected individuals.

**Electronic supplementary material:**

The online version of this article (doi:10.1186/s12985-017-0802-9) contains supplementary material, which is available to authorized users.

## Background

Human T-cell leukemia virus type 1 (HTLV-1) is a human retrovirus associated with two distinct types of disease: a malignancy of mature CD4+ T cells called adult T-cell leukemia-lymphoma (ATL) [1–3] and a chronic inflammatory central nervous system disease, HTLV-1-associated myelopathy/tropical spastic paraparesis (HAM/TSP) [4, 5]. In HTLV-1 infection, only approximately 5% of infected persons develop ATL [6] and another 0.25–4% develop HAM/TSP [7–10]. The majority of infected individuals remain lifelong asymptomatic carriers (ACs). However, since the treatment of both ATL and HAM/TSP remains highly unsatisfactory, the development of novel therapies for these intractable diseases is warranted.

Recently, the cancer/testis (CT) antigen NY-ESO-1, which was originally identified in esophageal cancer by serological expression cloning using autologous patient serum [11], has been reported to elicit both humoral and cellular immune responses in patients with ATL [12]. CT antigens are normally expressed only in the human germline cells in the testis, but are also expressed in various types of human cancers, making them an ideal target for cancer immunotherapy [13].

NY-ESO-1 is one of the most immunogenic CT antigens and has been a focus of investigation for the formulation of therapeutic vaccines [14]. In Japan, a phase I/II clinical trial using a novel redirected T-cell–based adoptive immunotherapy targeting NY-ESO-1 is currently underway for the treatment of relapse ATL after allogeneic hematopoietic stem cell transplantation. In addition, anti-NY-ESO-1 antibody was detected in patients with ATL and in ACs, indicating that an immune response against NY-ESO-1 is present in the course of natural HTLV-1 infection without malignancy [12]. However, the prognostic significance of anti-NY-ESO-1 in HTLV-1 infection is unclear.

The present study sought to clarify the relationship between humoral immune responses against NY-ESO-1 and the virological status of HTLV-1 infection. Plasma levels of anti-NY-ESO-1 autoantibody were measured and the possible correlation between levels of N-ESO-1 autoantibodies and virological parameters was assessed in samples derived from HTLV-1-infected individuals with different clinical status.

## Methods

### Patients

After obtaining written informed consent, plasma samples were collected from 130 patients with a clinical diagnosis of HAM/TSP, 53 patients with ATL, 51 ACs, and 22 uninfected normal controls (NCs). Diagnosis of HAM/TSP and ATL was based on the World Health Organization diagnostic criteria [15] and Shimoyama criteria [16], respectively.

### Real-time PCR analysis

To investigate the relationship between the expression levels of *tax* or *HBZ* mRNA and the antibody response against NY-ESO-1 in HTLV-1-infected individuals with different clinical status (i.e., HAM/TSP, ATL, and AC), we quantified the expression of *tax* or *HBZ* mRNA and the HTLV-1 PVL in peripheral blood mononuclear cells (PBMCs) by real-time PCR as described previously [17, 18].

### ELISA

Anti-NY-ESO-1 antibodies in plasma were quantified by an enzyme-linked immunosorbent assay (ELISA) using recombinant NY-ESO-1 protein (Additional file 1 Figure S1). Briefly, wells of a 96-well flat-bottom plate (MaxiSorp; Nunc, Roskilde, Denmark) were coated with 50 μL of purified NY-ESO-1 recombinant protein (1 μg/mL) and incubated overnight at 4 °C. Phosphate-buffered saline (PBS) was used as a control. The plates were washed three times with PBS containing 0.05% Tween 20 (PBS-T), and then blocked with a 1% skim milk in PBS-T (blocking buffer) at room temperature for 1 h. After washing five times with PBS-T, 50 μL of human plasma samples diluted 1/100 in blocking buffer was added to each NY-ESO-1- or PBS-coated well, and the plate was incubated for 1 h at room temperature. After washing three times with PBS-T, 50 μL of horseradish peroxidase-conjugated goat anti-human IgG F(ab’)_2_ (Jackson Immuno Research, West Grove, PA) diluted 1/10,000 in blocking buffer was added to the wells, and the plate was incubated for 1 h at room temperature. After washing five times with PBS-T, 50 μL of SureBlue™ TMB Microwell Peroxidase Substrate (Kirkegaard & Perry Laboratories, Gaithersburg, MD) was added to the wells, and the plate was incubated for 5 min at room temperature in the dark. The development reaction was stopped with 50 μL of 2 M sulfuric acid (H_2_SO_4_) and plates were read at 450 nm using a reference wavelength of 620 nm using a Multiskan™ FC automatic microplate reader (Thermo Fisher Scientific, Waltham, MA, USA). Each plasma sample was assayed three times in duplicate.

### Statistical analyses

To test for significant differences among four different groups of subjects (ATL, HAM/TSP, ACs and NCs), the Kruskal-Wallis test was employed. For multiple comparisons, we used the Scheffe’s F test to analyze statistical difference. The Mann-Whitney U test was used to compare data between two groups (i.e., aggressive and indolent ATL). Correlations between variables were examined using Spearman’s rank correlation analysis. The results shown are the mean ± SD where applicable. *P*-values <0.05 were considered statistically significant.

## Results

### Plasma levels of anti-NY-ESO-1 are significantly higher in patients with HAM/TSP than in patients with ATL and ACs

**Fig. 1:**
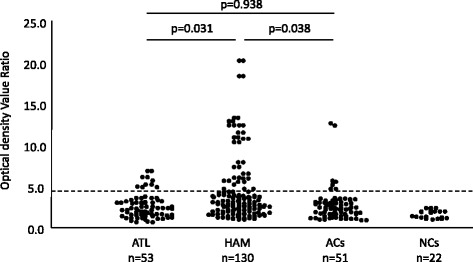
Detection of NY-ESO-1-specific antibodies in the plasma of HTLV-1-infected individuals with different clinical status Plasma anti-NY-ESO-1 antibody was detectable in 13.7% (7/51) of ACs, 29.2% (38/130) of patients with HTLV-1 associated myelopathy/tropical spastic paraparesis (HAM/TSP), and 18.9% (10/53) of patients with ATL. No humoral response against NY-ESO-1 was detected in 22 healthy uninfected controls (NCs). The plasma levels of anti-NY-ESO-1 was significantly higher in patients with HAM/TSP than in patients with ATL and ACs. NY-ESO-1 signals were evaluated by calculating the difference in absorbance values between the wells containing NY-ESO-1 and the wells containing PBS. Broken lines indicate the cut-off values (mean + 3 standard deviations of the control samples)

Plasma anti-NY-ESO-1 antibody was detectable in 13.7% (7/51) of ACs, 29.2% (38/130) of patients with HAM/TSP, and 18.9% (10/53) of patients with ATL. No humoral response against NY-ESO-1 was evident in 22 NCs (Fig. 1). Plasma levels of anti-NY-ESO-1 were significantly higher in patients with non-malignant chronic inflammatory disease HAM/TSP than in patients with malignant disease ATL or infected individuals without any symptoms (i.e., ACs) (Fig. 1).

### Plasma levels of anti-NY-ESO-1 in patients with indolent ATL are not significantly different from that in patients with aggressive ATL

**Fig. 2:**
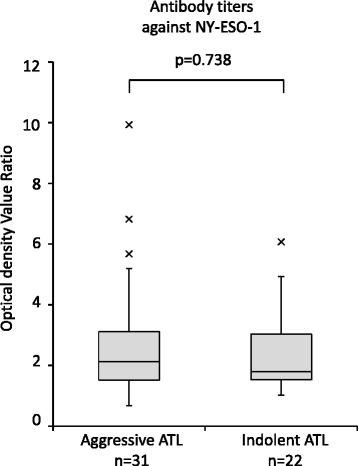
Plasma anti-NY-ESO-1 antibody titers between aggressive and indolent ATL Plasma anti-NY-ESO-1 antibody titers were compared between aggressive (acute or lymphoma type) and indolent (chronic or smoldering) clinical subtypes of ATL by ELISA. The mean optical density value ratios were not significantly different between aggressive and indolent ATL (*p* = 0.738 by Mann-Whitney U test)

Since previous studies suggested that NY-ESO-1-specific antibodies represent markers of recurrent or progressive disease in cancers that are not associated with viral infection, such as gastric cancer [19] and prostate cancer [20], we compared the levels of plasma anti-NY-ESO-1 between aggressive (acute or lymphoma type) and indolent (chronic or smoldering) clinical subtypes of ATL. There were no statistically significant differences in the levels of plasma anti-NY-ESO-1 between aggressive and indolent ATL (Fig. 2).

### Anti-NY-ESO-1 levels are not associated with PVL and expression of tax and HBZ mRNA among HTLV-1-infected individuals, regardless of clinical status

The antibody response to NY-ESO-1 did not correlate with *tax* and *HBZ* mRNA expression; moreover, the antibody response to NY-ESO-1 did not correlate with the PVL in any of the test groups (i.e., ATL, HAM/TSP and ACs) (Table 1 and Additional file 2 Figure S2), suggesting that virological status in HTLV-1-infected individuals does not affect the NY-ESO-1-specific immune responses.

**Table 1:** Results of rank correlation test between clinical status and virological parameters

	*HBZ* mRNA^a^		*tax* mRNA^b^		Proviral load^c^		*HBZ* mRNA/DNA^d^		*tax* mRNA/DNA^e^	
	r	p	r	p	r	p	r	p	r	p
ATL	0.045	0.764	−0.145	0.327	0.250	0.083	−0.120	0.411	−0.151	0.305
HAM/TSP	0.180	0.069	0.096	0.343	0.092	0.319	0.159	0.116	0.022	0.828
ACs	0.148	0.325	−0.011	0.942	−0.062	0.672	0.206	0.169	−0.012	0.934

Spearman’s rank correlation coefficients

^a^HTLV-1 *HBZ* mRNA load = value of HBZ / value of HPRT

^b^HTLV-1 *tax* mRNA load = value of tax / value of HPRT

^c^Proviral load: HTLV-1 *tax* copy number per cell

^d^ *HBZ* mRNA/DNA ratio = HTLV-1 HBZ mRNA load / Proviral load

^e^ *tax* mRNA/DNA ratio = HTLV-1 tax mRNA load / Proviral load

## Discussion

Previous studies have suggested that HTLV-1 infection is associated with an increase in the number of functional regulatory T-cells (Tregs). Since both the HTLV-1 transactivator Tax and the antisense-coded protein HBZ regulate FoxP3 expression at the level of transcription [21, 22], there is a significant increase in the number of HTLV-1 uninfected FoxP3 + CD4+ Tregs in HTLV-1-infected individuals. Tax suppresses FoxP3 expression in T cells [21], whereas HBZ induces Foxp3 expression, eventually resulting in an increase in the number of FoxP3+ T cells [22]. The size of CD4 + FoxP3+ population is also correlated with CCL22 produced by HTLV-1-infected T-cells [23]. Consequently, the increase in the number of HTLV-1-negative CD4 + FoxP3+ population determines the efficiency of T cell-mediated immune control of HTLV-1 and also the risk of developing HAM/TSP [24].

It has been well established that NY-ESO-1-specific CD4+ T-cell precursors exist at a relatively high frequency in healthy individuals, but are actively suppressed in the periphery by CD4 + CD25+ Treg cells [25, 26]. In other words, antigen-specific autoreactivity against NY-ESO-1 is a normal component of the CD4+ T cell population and is appropriately manipulated by Tregs. These findings suggest the possibility that the presence of anti-NY-ESO-1 antibodies is associated with the peripheral control of NY-ESO-1-specific T cells by Tregs, influenced by the virological status of HTLV-1 infection. We hypothesized that if anti-NY-ESO-1 antibodies are normally suppressed by Tregs, this suppression would be more efficient in HTLV-1-infected individuals. We therefore examined the levels of plasma anti-NY-ESO-1, and compared this to the *tax* or *HBZ* mRNA expression, and PVL.

Plasma levels of anti-NY-ESO-1 were significantly higher in patients with non-malignant chronic inflammatory HAM/TSP than in patients with malignant ATL and infected but asymptomatic individuals. In addition, there were no statistically significant differences in the levels of plasma anti-NY-ESO-1 between aggressive (acute or lymphoma type) and indolent (chronic or smoldering) clinical subtypes of ATL. Furthermore, anti-NY-ESO-1 levels were not associated with either PVL or the expression levels of *tax* and *HBZ* mRNA among HTLV-1-infected individuals, regardless of clinical status. These findings contradict the hypothesis that in vivo function of Tregs is associated with NY-ESO-1 seronegative status in healthy controls and highest plasma levels of anti-NY-ESO-1 in patients with HAM/TSP. If anti-NY-ESO-1 antibodies are normally suppressed by Tregs, this suppression would be more efficient in HTLV-1-infected individuals. The lack of correlation between levels of NY-ESO-1 autoantibodies and the virological parameters in samples derived from HTLV-1-infected individuals with different clinical status also contradicts the hypothesis.

Since the plasma level of anti-NY-ESO-1 antibodies was the highest in patients with HAM/TSP, it is more likely to be associated with the increased systemic immune activation and inflammatory profile of long-term HTLV-1-infected individuals. Recently, it was reported that NY-ESO-1-specific T cell responses were present in both recurrent hepatitis B virus (HBV)-related hepatocellular carcinoma (HCC) and non-recurrent HCC patients. However, HBV antigen-specific T cell responses were only present in patients with non-recurrent HCC and were completely absent in patients with recurrent HCC [27]. These results suggest that virus (i.e., HBV)-specific immune responses may prevent tumor recurrence, while NY-ESO-1-specific T cells may not. This may also be the case for HTLV-1 infection. Indeed, although previous reports correlated anti-NY-ESO-1 antibody titers with the clinical course of NY-ESO-1-positive malignancies [19, 20], we did not observe statistically significant differences in the levels of plasma anti-NY-ESO-1 between aggressive and indolent clinical subtypes of ATL.

## Conclusion

The data directly demonstrate the presence of autoreactive antibodies against CT antigen NY-ESO-1 in the peripheral blood of HTLV-1-infected individuals, irrespective of clinical status. Since NY-ESO-1 is one of the most immunogenic CT antigens recognized by both antibodies and T cells [11, 28, 29], detailed analyses are needed to investigate the prognostic value of NY-ESO-1 humoral immune response in HTLV-1 infection. It is also important to examine whether serum anti-NY-ESO-1 antibody titers correlate with the clinical status in individual patients over time. Before the anti-NY-ESO-1 immune response is used to influence the choice of immunotherapeutic approaches for patients with HTLV-1-associated diseases such as ATL and HAM/TSP, more information is required concerning the mechanisms underlying the generation of anti-NY-ESO-1 antibodies in HTLV-1-infected individuals.

## Additional files

Additional file 1: Figure S1. Preparation of His-NY-ESO-1 recombinant protein a. Sequence of NY-ESO-1 gene (NCBI Reference Sequence: NC_000023.11). b. Scheme of construction of pET-14b-NY-ESO-1 plasmid. The full-length NY-ESO-1 gene was amplified by PCR using the MT2 cell genome as a template. The three exons of NY-ESO-1 were separately amplified using primers matching the exon-termini. The DNA fragments of each exon were amplified by PCR using the following primer set: exon1: 5′-GGA ATT CCA TAT GCA GGC CGA AGG CCG GGG-3′ and 5′-AAC TCA AGC AGG CGG CTC TCC GGC CC-3′, exon2: 5′-CTA CCT CGC CAT GCC TTT CGC GA-3′ (exon2-for primer) and 5′-ATA GTC AGT ATG TTG CCG GAC ACA-3′, exon3: 5′- CCG ACT GAC TGC TGC AGA CCA-3′ and 5′-TCG CGG ATC CTT AGC GCC TCT GCC CTG AGG GAG G-3′ (exon3-rev primer). To combine exon2 and exon3 fragments, the joined exon2–3 DNA fragment was amplified by an overlap PCR using exon2-for and exon3-rev as primers and the generated each two DNA fragments as template. Exon1 and exon2–3 DNA fragments were phosphorylated with T4 polynucleotide kinase (Takara Bio Inc., Shiga, Japan). The exon1 fragment was digested with *Nde*I, and the exon2–3 fragment was digested with *Bam*HI, and cloned into *Nde*I- and *Bam*HI-digested pET-14b. This plasmid was used for transformation of *Escherichia coli* BL21 (DE3). c. Amplified three exons of NY-ESO-1 were subjected to agarose gel electrophoresis. d. His-NY-ESO-1 recombinant protein was purification using Ni Sepharose 6 Fast Flow (GE Healthcare Japan, Tokyo, Japan). (PDF 57 kb)

Additional file 2: Figure S2. Results of rank correlation test between anti-NY-ESO-1 antibody titers and virological parameters in HTLV-1-infected individuals with different clinical status. The antibody response to NY-ESO-1 did not correlate with both *HBZ* (a) and *tax* (b) mRNA expression and HTLV-1 proviral load (c). To test whether higher *HBZ* or *tax* mRNA levels reflect higher proviral load, we adjusted the *HBZ* or *tax* mRNA load (i.e. value of *tax* or *HBZ*/value of HPRT) by the HTLV-1 proviral load (i.e. HTLV-1 tax copy number per cell). As a result, the antibody response to NY-ESO-1 did not correlate with both *tax* (d) and *HBZ* (e) mRNA expression per provirus. Spearman’s rank correlation coefficient (r) and level of significance (p) are indicated within each graph. (PDF 134 kb)

## Abbreviations

ACs: Asymptomatic carriers
ATL: Adult T-cell leukemia
HAM/TSP: HTLV-1-associated myelopathy/tropical spastic paraparesis
HPRT: Hypoxanthine phosphoribosyltransferase
HTLV-1: Human T-cell leukemia virus type-1
NCs: Normal uninfected healthy controls
PVL: Proviral load
