# Supplementary material for: Prevalence of plasma autoantibody against cancer testis antigen NY-ESO-1 in HTLV-1 infected individuals with different clinical status
Source: Virol J. 2017 Jul 17;14:130. doi: 10.1186/s12985-017-0802-9 (PMC5512893; doi:10.1186/s12985-017-0802-9)
Supplement: Supplementary file 1 — Preparation of His-NY-ESO-1 recombinant protein a. Sequence of NY-ESO-1 gene (NCBI Reference Sequence: NC_000023.11). b. Scheme of construction of pET-14b-NY-ESO-1 plasmid. The full-length NY-ESO-1 gene was amplified by PCR using the MT2 cell genome as a template. The three exons of NY-ESO-1 were separately amplified using primers matching the exon-termini. The DNA fragments of each exon were amplified by PCR using the following primer set: exon1: 5′-GGA ATT CCA TAT GCA GGC CGA AGG CCG GGG-3′ and 5′-AAC TCA AGC AGG CGG CTC TCC GGC CC-3′, exon2: 5′-CTA CCT CGC CAT GCC TTT CGC GA-3′ (exon2-for primer) and 5′-ATA GTC AGT ATG TTG CCG GAC ACA-3′, exon3: 5′- CCG ACT GAC TGC TGC AGA CCA-3′ and 5′-TCG CGG ATC CTT AGC GCC TCT GCC CTG AGG GAG G-3′ (exon3-rev primer). To combine exon2 and exon3 fragments, the joined exon2–3 DNA fragment was amplified by an overlap PCR using exon2-for and exon3-rev as primers and the generated each two DNA fragments as template. Exon1 and exon2–3 DNA fragments were phosphorylated with T4 polynucleotide kinase (Takara Bio Inc., Shiga, Japan). The exon1 fragment was digested with NdeI, and the exon2–3 fragment was digested with BamHI, and cloned into NdeI- and BamHI-digested pET-14b. This plasmid was used for transformation of Escherichia coli BL21 (DE3). c. Amplified three exons of NY-ESO-1 were subjected to agarose gel electrophoresis. d. His-NY-ESO-1 recombinant protein was purification using Ni Sepharose 6 Fast Flow (GE Healthcare Japan, Tokyo, Japan). (PDF 57 kb) [file 12985_2017_802_MOESM1_ESM.pdf]

A Sequence of NY-ESO-1 gene (NCBI Reference Sequence: NC_000023.11)

```
ATCCTCGTGGGCCCTGACCTTCTCTCTGAGAGCCGGGCAGAGGCTCCGGAGCCATGCAGGCCGAAGGCCG
GGGCACAGGGGGTTCGACGGGCGATGCTGATGGCCCAGGAGGCCCTGGCATTCCTGATGGCCCAGGGGGC
AATGCTGGCGGCCCAGGAGAGGCGGGTGCCACGGGCGGCAGAGGTCCCCGGGGCGCAGGGGCAGCAAGGG   Exon1
CCTCGGGGCCGGGAGGAGGCGCCCCGCGGGGTCCGCATGGCGGCGCGGCTTCAGGGCTGAATGGATGCTG
CAGATGCGGGGCCAGGGGGCCGGAGAGCCGCCTGCTTGAGTTGTATTCTGTTCTGTTCTGTTCTGTTCTG
TTCTGACAGTTCTGGTGGCGAGGTGGGGGCCGGGAGATGGGGAGGGCAGGGCCAGGTGGGGGAGGAGGCG
GGGGAGATGCGAGTAAGTGGTTGGGTGGGGGTGGGGGTGGGGGTTGGGAGGTGGGGATATGAGAGGCCAG
CTGCGAGGAGGGGAGGAGGTAAGGGCTGGGAGGTAGAGGGGGTGGGGGTGGGGGAGAGGGGCTGGGTGAT
TGGGTGAGGGGCACCGGGTCATGCGGGGAGGGGAGCAGGGGCCAGGAGATAGGGGGACCCAGGTGTAGGA
GGGGATCAGGAGGTGGGGGGAAGGGGGCAAGATGGGAGCGGGTGGCGGGAGGTGGTTGGGATGGAGGGTC
GAGTGATGAGGAGGGGGGCCGGGTGATGCCACAGGGGGCAGGGTGGGGAGGGGTCTTGTAAGGGCATGGAG
GAGTCAGCTTGGGGTGCAGCAAAGGAAAGTGGGGAACCAAGACAGCTGGGTGGGGTGTGCTACTGGGCCA
GTGCCTGGGGAGACAGCCTGGGGGGGATGGGCTGGGCAATCCGGGGTAGGGGGGAGGGGTGGGGGAGTGG
GGGAGGGGGAAGCTGGCCTGGGGTAAGCAGCAGGCCGGAGCCACAGGCCAAGTCCTAGGAGAGGATGCCT
TAACTGGGCCCCCACCAGCTACCTCGCCATGCCTTTCGCGACACCCATGGAAGCAGAGCTGGCCCGCAGG
AGCCTGGCCCAGGATGCCCCACCGCTTCCCGTGCCAGGGGTGCTTCTGAAGGAGTTCACTGTGTCCGGCA   Exon2
ACATACTGACTATGTCAGTTCAGGACCAGGACAGGGATGGGGCCTGGGTGGGTGGCGGTCACAGCGTGGC
AGGGTGGGGGCTGGGATCCGCCTACACCCCACGGTCAGGCTGCTAGAAACCTGGGAACACCCCAGCACAG
GGTCTCAGAACAGAGACCTGGTACACCAGGCCCGCCGCCACCCGAGGGAGCCCAGGGAGATGGGTGCAGA
GGTGTCGCCTTTAACGTGATGTTCTCTGCCCCTCACATTTAGCCGACTGACTGCTGCAGACCACCGCCAA
CTGCAGCTCTCCATCAGCTCCTGTCTCCAGCAGCTTTCCCTGTTGATGTGGATCACGCAGTGCTTTCTGC   Exon3
CCGTGTTTTTGGCTCAGCCTCCCTCAGGGCAGAGGCGCTAAGCCCAGCCTGGCGCCCCTTCCTAGGTCAT
GCCTCCTCCCCTAGGGAATGGTCCCAGCACGAGTGGCCAGTTCATTGTGGGGGCCTGATTGTTTGTCGCT
GGAGGAGGACGGCTTACATGTTTGTTTCTGTAGAAAATAAAACTGAGCTACGA
```

B Scheme of construction of pET-14b-NY-ESO-1 plasmid

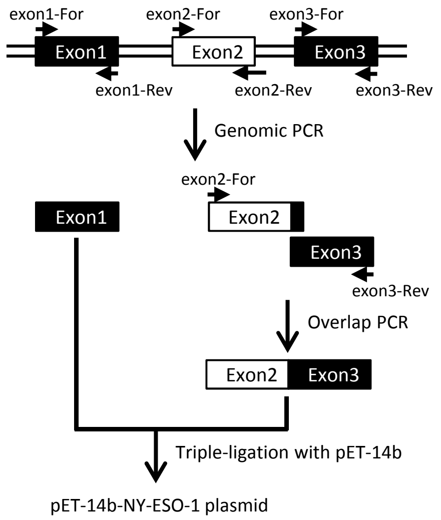

C Amplification of DNA fragments of exons

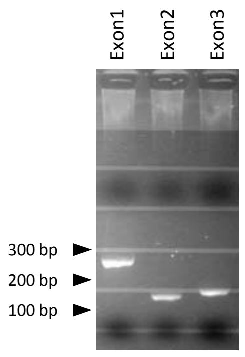

DNA size
Exon1: 269 bp
Exon2: 135 bp
Exon3: 139 bp

D Purification of His-NY-ESO-1 protein

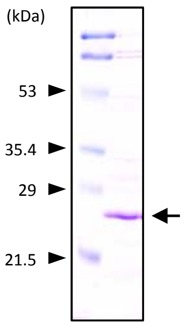

Supplementary Figure 1
