# Supplementary figures and images for: Prevalence of plasma autoantibody against cancer testis antigen NY-ESO-1 in HTLV-1 infected individuals with different clinical status
Source: Virol J. 2017 Jul 17;14:130. doi: 10.1186/s12985-017-0802-9 (PMC5512893; doi:10.1186/s12985-017-0802-9)

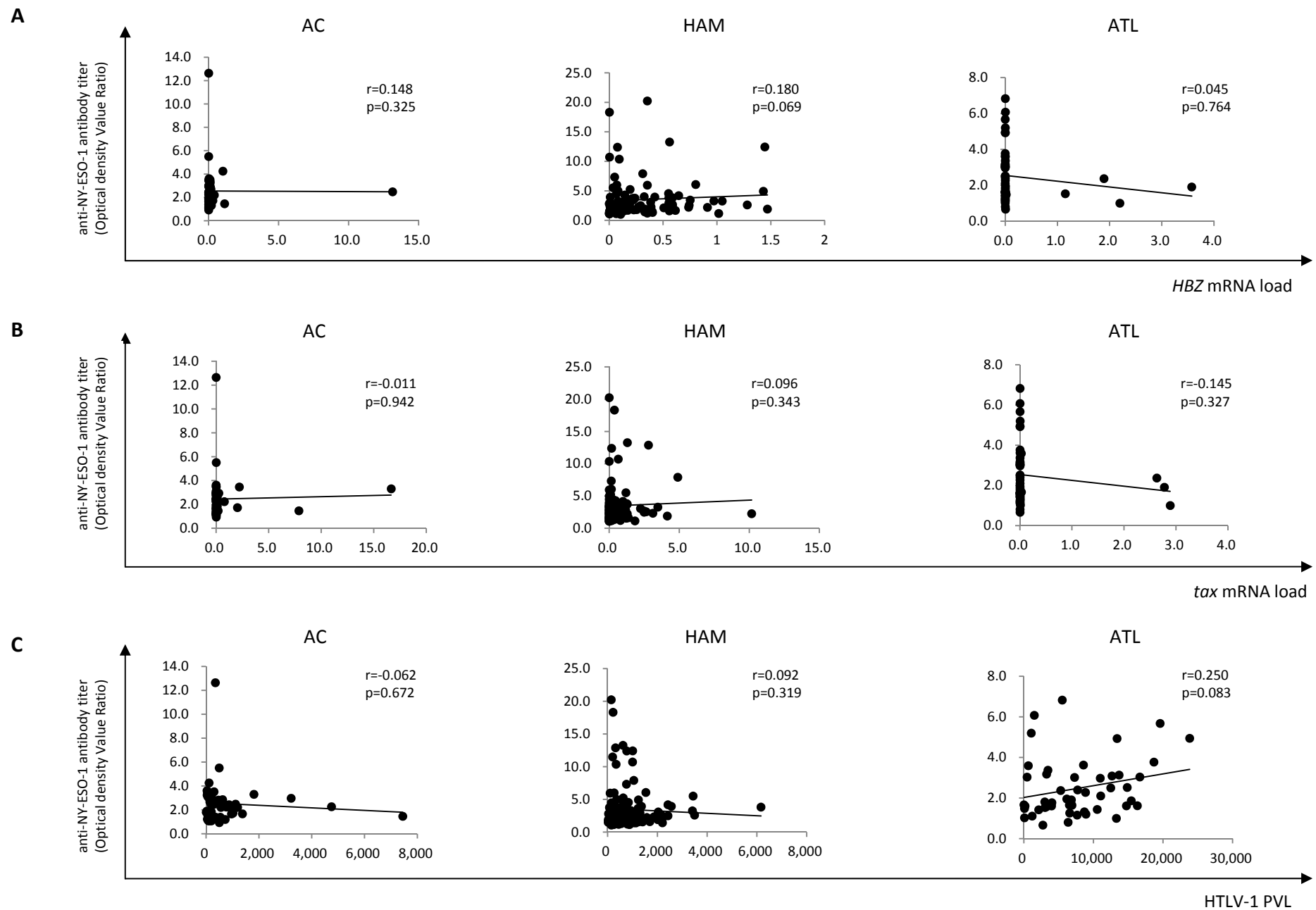

Supplementary Figure 2

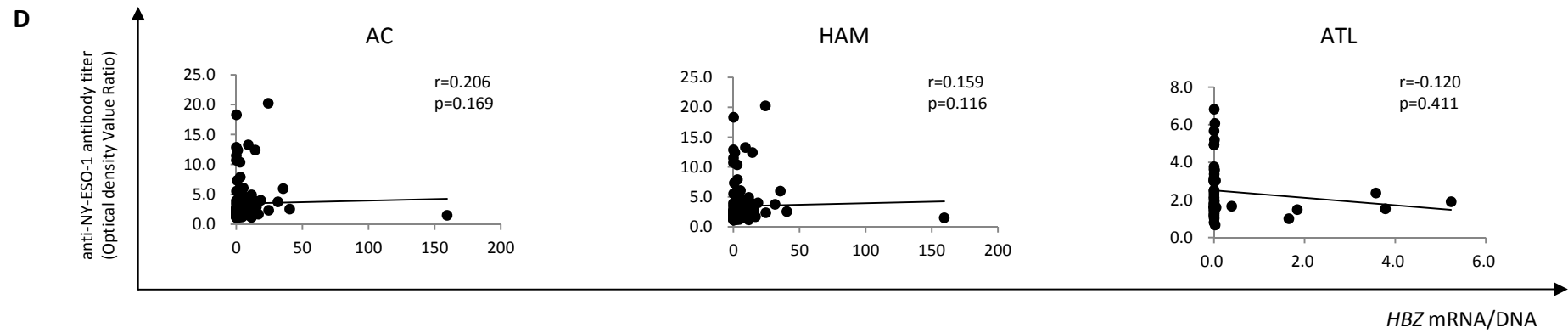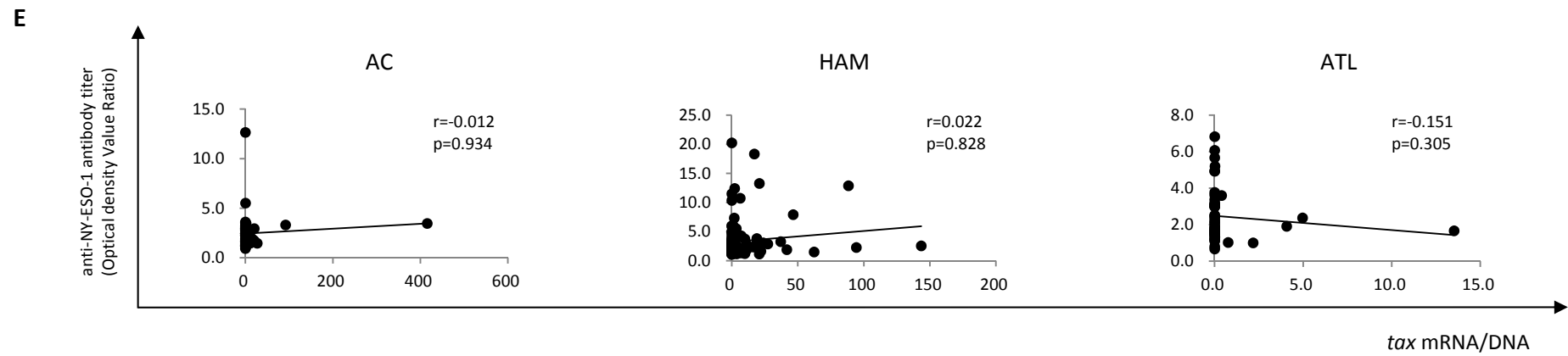

Supplementary Figure 2

Supplement: Supplementary file 2 — Results of rank correlation test between anti-NY-ESO-1 antibody titers and virological parameters in HTLV-1-infected individuals with different clinical status. The antibody response to NY-ESO-1 did not correlate with both HBZ (a) and tax (b) mRNA expression and HTLV-1 proviral load (c). To test whether higher HBZ or tax mRNA levels reflect higher proviral load, we adjusted the HBZ or tax mRNA load (i.e. value of tax or HBZ/value of HPRT) by the HTLV-1 proviral load (i.e. HTLV-1 tax copy number per cell). As a result, the antibody response to NY-ESO-1 did not correlate with both tax (d) and HBZ (e) mRNA expression per provirus. Spearman’s rank correlation coefficient (r) and level of significance (p) are indicated within each graph. (PDF 134 kb) [file 12985_2017_802_MOESM2_ESM.pdf]
